# Clinical utility of rapid on-site evaluation of brush cytology during bronchoscopy using endobronchial ultrasound with a guide sheath

**DOI:** 10.1038/s41598-024-72138-z

**Published:** 2024-09-12

**Authors:** Kazuhiro Nishiyama, Kei Morikawa, Shotaro Kaneko, Makoto Nishida, Aya Matsushima, Yoshihiro Nishi, Yu Numata, Yusuke Shinozaki, Hajime Tsuruoka, Hirotaka Kida, Hiroshi Handa, Naoki Shimada, Chie Okawa, Nobuyuki Ohike, Junki Koike, Masamichi Mineshita

**Affiliations:** 1 Department of Respiratory Medicine, St. Marianna University School of Medicine, Kawasaki, Japan; 2 Department of Pathology, St. Marianna University Hospital, Kawasaki, Japan; 3 Department of Pathology, St. Marianna University School of Medicine, Kawasaki, Japan

**Keywords:** Rapid on-site evaluation, Endobronchial ultrasound with a guided sheath, Peripheral lung lesions, Brushing, Lung cancer, Medical research, Respiratory tract diseases

## Abstract

Previous studies have shown that rapid on-site evaluation (ROSE) improves the diagnostic yield of bronchoscopy using endobronchial ultrasound with a guide sheath (EBUS-GS) for peripheral pulmonary lesions (PPL). While ROSE of imprint cytology from forceps biopsy has been widely discussed, there are few reports on ROSE of brush cytology. This study investigated the utility of ROSE of brush cytology during bronchoscopy. We retrospectively analyzed data from 214 patients who underwent bronchoscopy with EBUS-GS for PPL. The patients in the ROSE group had significantly higher diagnostic sensitivity through the entire bronchoscopy process than in the non-ROSE group (96.8% vs. 83.3%, P = 0.002). The use of ROSE significantly increased the sensitivity of brush cytology with Papanicolaou staining (92.9% vs. 75.0%, P < 0.001). When ROSE was sequentially repeated on brushing specimens, initially negative ROSE results converted to positive in 79.5% of cases, and the proportion of specimens with high tumor cell counts increased from 42.1 to 69.0%. This study concludes that ROSE of brush cytology improves the diagnostic accuracy of bronchoscopy and enhances specimen quality through repeated brushing.

## Introduction

Bronchoscopy is a key method for diagnosing peripheral pulmonary lesions (PPL). In bronchoscopic specimen collection from PPL, the approach to the lesion and the method of specimen collection are crucial. Various techniques for approaching lesions have been examined, including electromagnetic navigation bronchoscopy, virtual bronchoscopic navigation, radial endobronchial ultrasound (R-EBUS), ultrathin bronchoscopy, and guide sheath (GS)^1–6^. Bronchoscopy using endobronchial ultrasound with a guide sheath (EBUS-GS) is commonly performed to approach PPL, with forceps biopsy being a commonly used method for specimen collection. Other methods, often combined with forceps biopsy, include brushing, washing, and cryobiopsy^7^.

In recent years, with the advent of molecular-targeted drugs and immune checkpoint inhibitors for lung cancer therapy, testing for genetic mutation and programmed cell death ligand-1 (PD-L1) of bronchoscopic specimens has become increasingly important. Next-generation sequencing (NGS), which can detect multiple genetic mutations simultaneously, is often used for genetic mutation testing^8^. Conventional NGS testing requires a certain tissue size and tumor cell content^9^. Against this background, obtaining large tissue specimens with high tumor cell content during bronchoscopy has become more desirable, particularly with the widespread use of cryobiopsy^10–12^. However, it is now possible to perform NGS using cytological specimens^13–15^. Furthermore, it has been suggested that the level of PD-L1 in cytological specimens correlates strongly with that in tissue specimens^16–18^. Therefore, cytological specimens obtained during bronchoscopy are becoming increasingly important. Among cellular specimens, brush cytology has been reported to have high diagnostic accuracy^19^; hence, brush cytology has received renewed attention in recent years.

Rapid on-site evaluation (ROSE) is used to confirm the presence of tumor cells in cytological specimens, and its utility in bronchoscopy has been studied using endobronchial ultrasound-guided transbronchial needle aspiration (EBUS-TBNA). Studies suggest that ROSE improves the diagnostic yield of EBUS-TBNA and decreases the number of biopsies required^20–22^. Similar findings have been reported for bronchoscopy of PPL^23–28^. When ROSE is performed during bronchoscopy with EBUS-GS for PPL, two types of cytology specimens can be evaluated: forceps biopsy imprint cytology and brush cytology. While there are many reports on ROSE of forceps biopsy imprint cytology^23–26^, there are few reports on ROSE of brush cytology^27,28^. We investigated the improvement in diagnostic yield and changes in ROSE results with repeated brushing when ROSE was performed on brush cytology during bronchoscopy using EBUS-GS for PPL.

## Methods

### Patients

This retrospective study was approved by the Ethics Committee of the St. Marianna University School of Medicine on October 26, 2023, with approval number 6224. The data used in this study were anonymized; therefore, the requirement for informed consent was waived by the Ethics Committee of the St. Marianna University School of Medicine and the study was compliant with The Declaration of Helsinki.

We included patients who underwent bronchoscopy for PPL at St. Marianna University Hospital between October 2020 and October 2022. PPL was defined as a nodule surrounded by the pulmonary parenchyma. Patients underwent tumor marker assessments and chest computed tomography (CT) scans before bronchoscopy. Bronchoscopy was performed only when deemed necessary for diagnostic purposes, which typically included cases with a high suspicion of malignancy based on prior assessments. Patients who underwent endobronchial biopsy or did not undergo EBUS-GS were excluded.

After a one-year study of medical records following bronchoscopy, the final diagnosis was defined as malignant for cases with a pathologic diagnosis of malignancy and benign for other cases.

### Biopsy procedure

Prior to bronchoscopy, a high-resolution chest CT scanner was employed to identify the bronchi leading to the lesions. The sequence of bronchoscopies was determined randomly. ROSE was conducted for all bronchoscopies where cytologists were available. ROSE was omitted when cytologists were not available.

Bronchoscopy was performed using a combination of a bronchoscope (BF-1T260 or BF-P260F; Olympus, Tokyo, Japan), an R-EBUS probe (20 MHz mechanical radial type, UM-S20-17S; Olympus), and a GS kit (K-201 or K-203). During the procedure, patients were administered intravenous midazolam and fentanyl for mild sedation and analgesia, with the bronchoscope inserted either orally or nasally. The GS containing the R-EBUS probe was introduced through the bronchoscope's working channel and guided to the lesion site. EBUS images were categorized into three types: "within," "adjacent to," and "invisible." After evaluating the fluoroscopic and EBUS images, the R-EBUS probe was removed, and brushing and forceps biopsy were performed. In the ROSE group, the immediate results of ROSE from the glass slides produced by brushing were communicated to the examiner. If the EBUS image was classified as "adjacent to" or "invisible," the position of the R-EBUS probe was adjusted or the bronchi were re-navigated to achieve a "within" classification. The number of brushings, forceps biopsies, and procedural adjustments were at the examiner’s discretion. There was no standardized number of brushings or forceps biopsies per patient; the number varied depending on the individual case and clinical judgment.

### Processing of specimens

In the ROSE group, brushing specimens were transferred onto four glass slides. One slide was rapidly air-dried and stained with Cyto-Quick stain (Cyto-Quick; Muto Pure Chemicals, Tokyo, Japan), whereas the other three slides were fixed in 95% alcohol. The Cyto-Quick-stained slide specimens were microscopically evaluated by a cytologist in the same bronchoscopy laboratory. The alcohol-fixed slides were Papanicolaou-stained after bronchoscopy and microscopically evaluated by a cytologist. In the non-ROSE group, brushing specimens were transferred onto two glass slides and fixed in 95% alcohol. These slides were also Papanicolaou-stained and evaluated after bronchoscopy, similar to the ROSE group. Four glass slides were prepared for each brushing in the ROSE group, whereas only two were prepared for the non-ROSE group. In both groups, the brush was tapped on the glass slides to scatter the cells while transferring cells from the brush to the slides. Forceps biopsy specimens were placed in 10% formalin and stained with hematoxylin–eosin after bronchoscopy for histological evaluation.

### Cytological specimen diagnosis

Cytological diagnosis was performed according to the World Health Organization reporting system for lung cytopathology^29,30^. Lung cytopathology specimens were classified into five categories: insufficient/inadequate/non-diagnostic, benign, atypical, suspicious for malignancy, and malignant. Among these five categories, specimens classified as suspicious for malignancy or malignant were defined as positive, while all others were defined as negative. Several pathologists and cytologists confirmed the cytological diagnosis and evaluation at our hospital.

**Fig. 1:**
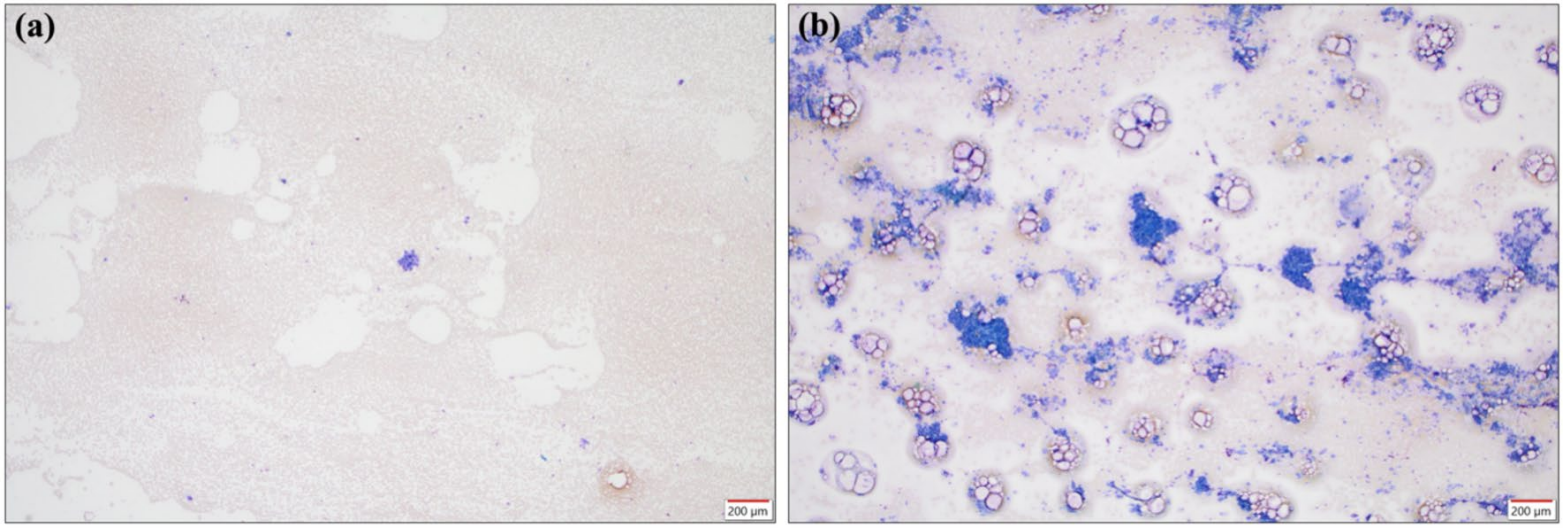
Distinction between Class 3 and Class 4: (**a**) Class 3, (**b**) Class 4. Samples were stained with Cyto-Quick and examined under a microscope at × 40 magnification. Class 3 showed scattered clusters, with 1–2 clusters per field of view and 7 clusters per glass slide. Class 4 showed multiple clusters per field of view and numerous clusters per glass slide.

To evaluate tumor cell counts, ROSE results were classified into four categories: no malignant findings (class 1), atypical cells or suspected malignant cells (class 2), sufficient malignant cells (class 3), and many malignant cells (class 4). The sufficient malignant cells were based on the Bethesda system in thyroid cytopathology, requiring a minimum of 6–7 clusters^31^. The distinction between class 3 and class 4 is shown in Fig. 1. Classes 1 and 2 were defined as ROSE-negative, while classes 3 and 4 were defined as ROSE-positive. The ROSE classification was determined for each brushing, and the highest observed class was designated as the ROSE diagnosis.

### Statistical analysis

Statistical analyses were performed using EZR (Saitama Medical Center, Jichi Medical University, Saitama, Japan), a graphical user interface for R (The R Foundation for Statistical Computing, Vienna, Austria)^32^. Continuous variables were expressed as median (range), and differences were compared using the Mann–Whitney U test. Categorical variables were expressed as percentages, and differences were compared using Fisher's exact test. In this study, "sensitivity" is specifically defined as the diagnostic sensitivity for malignant lesions. Multivariable logistic regression was used to control for potential confounding effects of lesion size, lesion location, number of specimens, and EBUS images to compare the sensitivity between the two groups. Statistical significance was set at P < 0.05.

## Results

**Fig. 2:**
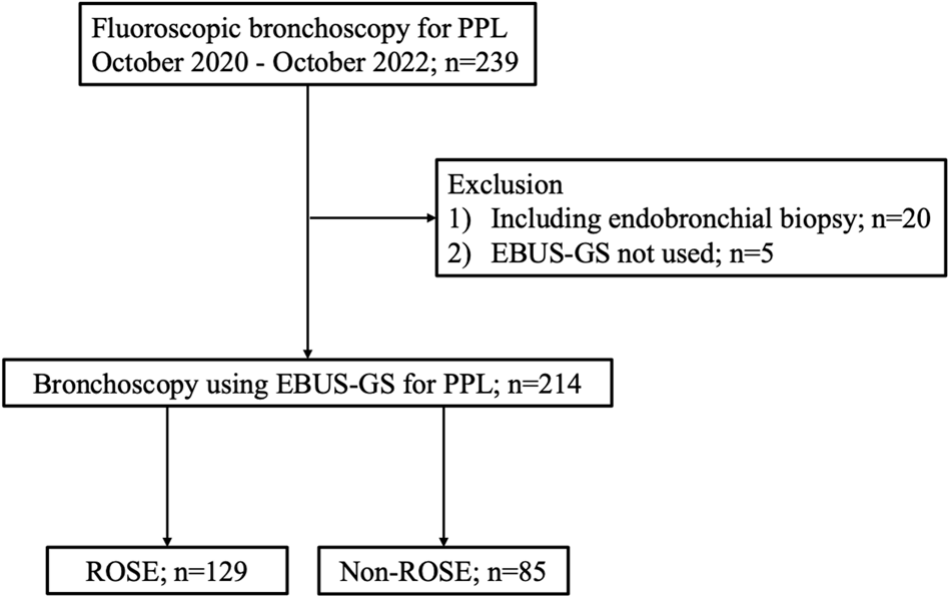
Flow chart of patients enrolled in the study. PPL, peripheral pulmonary lesions; EBUS-GS, endobronchial ultrasound with a guide sheath; ROSE, rapid on-site evaluation.

239 patients underwent bronchoscopy for PPL between October 2020 and October 2022. Of those, 20 patients underwent endobronchial biopsy and five did not undergo EBUS-GS were excluded. Ultimately, 214 patients were included in the study, with 129 patients in the ROSE group and 85 in the non-ROSE group (Fig. 2).

Baseline characteristics and bronchoscopy details using EBUS-GS are summarized in Table 1. There were no statistically significant differences in lesion size between the ROSE group (median: 34 mm, range: 14–130 mm) and the non-ROSE group (median: 31 mm, range: 9–83 mm; P = 0.076). Lesion locations were similarly distributed among the groups. Brushing was performed on all patients, and forceps biopsy was performed on approximately 90% of patients in both groups. There were no significant differences in the number of specimens obtained from brushings between the ROSE group (median: 3, range: 2–9) and the non-ROSE group (median: 3, range: 2–7; P = 0.789). Notably, the ROSE group had a significantly higher number of cases with “within” EBUS images than the non-ROSE group (89.9% vs. 78.8%, P = 0.029). The final diagnosis of malignancy was significantly higher in the ROSE group than in the non-ROSE group (89.9% vs. 84.7%, P < 0.001).

**Table 1:** Baseline characteristics and bronchoscopy details using EBUS-GS.

**Characteristics**	**ROSE**	**Non-ROSE**	**P value**
**Patients, n**	129	85	
**Age [years], median (range)**	72 (38–86)	75 (27–89)	0.072
**Gender, n (%)**			
Male	77 (59.7)	42 (49.4)	0.160
Female	52 (40.3)	43 (50.6)	
**Lesion size [mm], median (range)**	34 (14–130)	31 (9–83)	0.076
≦ 30 mm, n (%)	54 (41.9)	40 (47.1)	0.484
> 30 mm, n (%)	75 (58.1)	45 (52.9)	
**Lesion location, n (%)**			
RUL and LUS	67 (51.9)	50 (58.8)	0.613
RML and lingula	14 (10.9)	7 (8.2)	
RLL and LLL	48 (37.2)	28 (32.9)	
**Inspection Contents, n (%)**			
Brushing	129 (100.0)	82 (100.0)	1.000
Forceps biopsy	115 (89.1)	74 (87.1)	0.668
**Number of specimens, median (range)**			
Brushing	3 (2–9)	3 (2–7)	0.789
Forceps biopsy	3 (1–8)	3 (1–8)	0.766
**EBUS image, n (%)**			
Within	116 (89.9)	67 (78.8)	0.029*
Not within	13 (10.1)	18 (21.2)	
Adjacent to	11 (8.5)	11 (12.9)	
Invisible	2 (1.6)	7 (8.2)	
**Final diagnosis, n (%)**			
Benign	3 (2.3)	13 (15.3)	< 0.001*
Malignant	126 (96.9)	72 (84.7)	
Adenocarcinoma	88 (68.2)	48 (56.5)	
Squamous carcinoma	21 (16.3)	12 (14.1)	
NSCLC	7 (5.4)	5 (5.9)	
Small cell carcinoma	3 (2.3)	1 (1.2)	
LCNEC	1 (0.8)	2 (2.4)	
Malignant lymphoma	1 (0.8)	2 (2.4)	
Metastatic malignancy	3 (2.3)	2 (2.4)	
Adenosquamous carcinoma	1 (0.8)		
Large cell carcinoma	1 (0.8)		

*EBUS-GS* endobronchial ultrasound with a guided sheath, *ROSE* rapid on-site evaluation, *RUL* right upper lobe, *RML* right middle lobe, *RLL* right lower lobe, *LUS* left upper segment, *LLL* left lower lobe, *EBUS* endobronchial ultrasound, *NSCLC* non-small cell lung cancer, *LCNEC* large-cell neuroendocrine carcinoma.

Table 2 shows the sensitivity of bronchoscopy in both groups. The overall sensitivity of the bronchoscopy results was significantly higher in the ROSE group than in the non-ROSE group (96.8% vs. 83.3%, P = 0.002). Multivariate analysis confirmed this significant difference (P = 0.004). Specifically, the sensitivity of Papanicolaou stain evaluation of brushing was significantly higher in the ROSE group than in the non-ROSE group (92.9% vs. 75.0%, P < 0.001). Although the sensitivity of forceps biopsy was higher in the ROSE group than in the non-ROSE group, this difference was not statistically significant (87.6% vs. 78.8%, P = 0.137).

**Table 2:** Sensitivity of bronchoscopy in both groups.

**Sensitivity, n/n (%)**	**ROSE**	**Non-ROSE**	**P value**	**Multivariate**		
				**OR**	**95% CI**	**P value**
Bronchoscopy	122/126 (96.8)	60/72 (83.3)	0.002*	8.55	1.96–37.30	0.004*
Brushing	117/126 (92.9)	54/72 (75.0)	< 0.001*	6.06	2.11–17.40	< 0.001*
Forceps biopsy	99/113 (87.6)	52/66 (78.8)	0.137	2.27	0.88–5.83	0.089

*ROSE* rapid on-site evaluation, *OR* odds ratio, *CI* confidence interval.

**Fig. 3:**
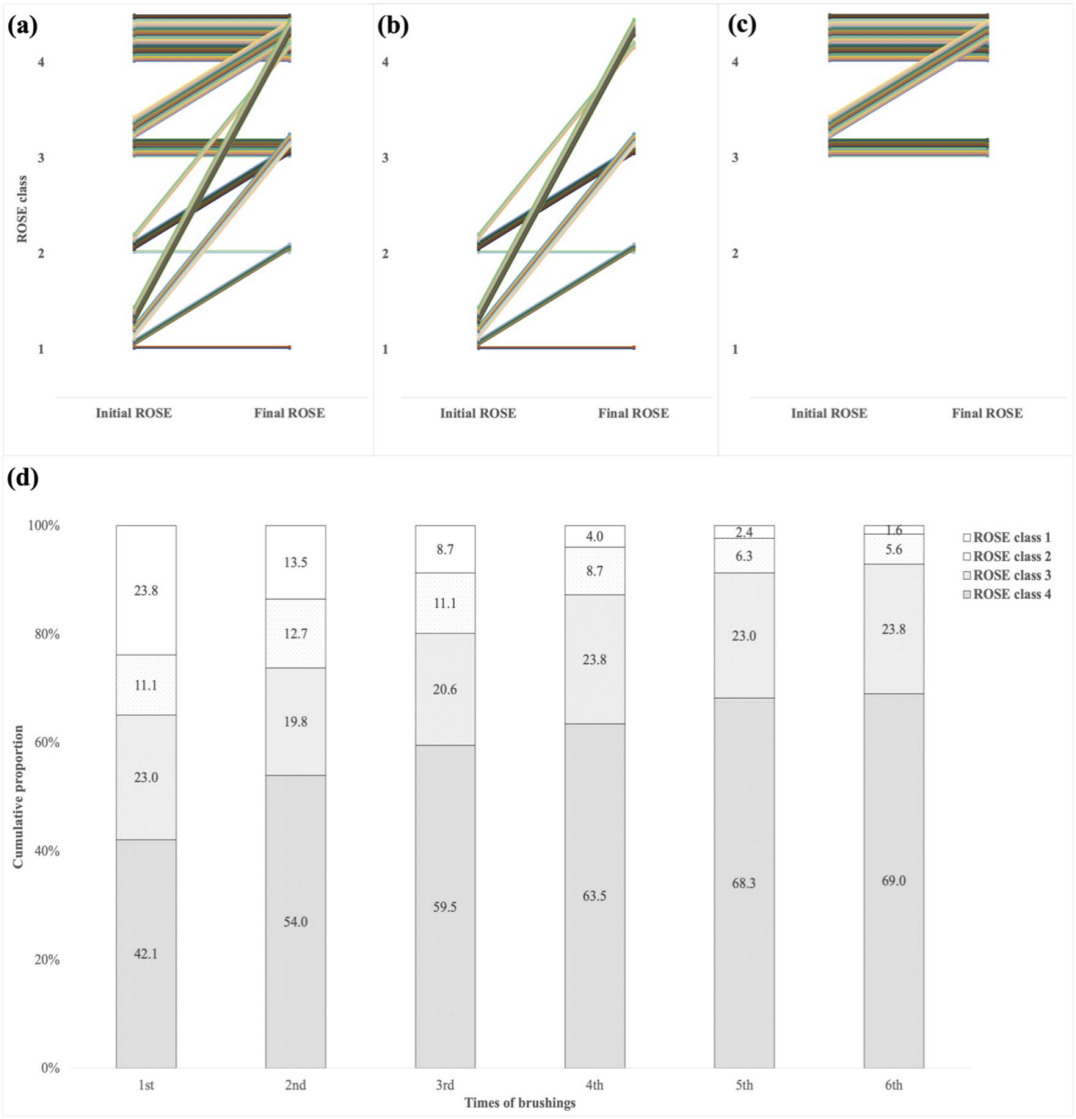
The change from the initial ROSE to the final ROSE diagnosis. (**a**) All cases and cases with initial (**b**) ROSE-negative or (**c**) ROSE-positive results are shown. (**d**) The cumulative proportion in the best ROSE result is analyzed by the number of brushings. ROSE, rapid on-site evaluation.

Figure 3 illustrates the change in ROSE results among the 126 patients in the ROSE group with a final diagnosis of malignancy. Initially, 44 patients (34.9%) had negative ROSE results. Among these, 35 patients (79.5%) converted to positive ROSE results after repeated brushing (Fig. 3b). Of these 35 patients, 20 achieved positive results with repeated brushing at the same site, nine by changing the bronchial branch, and six by adjusting the depth of brushing. Conversely, 82 patients (65.1%) initially had positive ROSE results. The initial ROSE result was classified as class 3 in 29 patients, and 16 of these patients (55.2%) improved to class 4 with repeated brushing (Fig. 3c). The proportion of patients whose ROSE results were classified as Class 4 was 42.1% at the first brushing. This proportion increased with each additional brushing, reaching a cumulative proportion of 69.0% by the sixth brushing (Fig. 3d).

## Discussion

In this study, ROSE of brush cytology improved the sensitivity of bronchoscopy using EBUS-GS for PPL. Repeated brushing procedures change the ROSE result from negative to positive and increased tumor cell counts in brushing cytology in some cases.

ROSE of brush cytology has proven to be a valuable tool in bronchoscopy for PPL. We advocate for the routine incorporation of ROSE in brush cytology into clinical practice to enhance the accuracy and efficiency of bronchoscopic diagnoses. Moreover, ROSE has been shown to increase the sensitivity of brushing. By allowing real-time evaluation of brushing specimens, ROSE enables the examiner to repeat the procedure until sufficient specimens are collected, thereby improving diagnostic sensitivity.

The rationale for this is that there were many cases wherein ROSE diagnosis changed from negative to positive during bronchoscopy. Of the 44 initially ROSE-negative cases, 35 (79.5%) converted to a ROSE-positive diagnosis. Of these, 20 tested positive after repeated brushing of the same site. Fifteen of 20 patients had adenocarcinomas. Kurimoto et al. (2004) reported that tumors from which specimens cannot be obtained, even when EBUS images indicate they are “within,” are often adenocarcinomas^1^. Because adenocarcinomas are often covered by tracheal epithelium, specimen collection may be impossible without repeated brushing and destruction of the tracheal epithelium. For patients with suspected adenocarcinoma based on chest CT imaging or tumor markers prior to bronchoscopy, repeated specimen collection from the same site is recommended. In contrast, the brushing position was changed during bronchoscopy in 15 patients. Of these, nine had changes in the bronchial branches, and six changed the depth of the brushing position. It is speculative, but the high number of "within" in the EBUS images in the ROSE group may have been caused by these changes in the brushing position. Patients in whom the bronchial branch changed had another bronchial branch, which was a candidate for specimen collection based on the chest CT imaging prior to bronchoscopy. All patients for whom the depth of the brushing position was changed had lesions measuring greater than 30 mm. We believe it is crucial to conduct detailed confirmation of the lesion through chest CT imaging or other modalities prior to bronchoscopy and to adapt the procedure based on the results of ROSE. In detail, if the initial ROSE result is negative, we suggest modifying the bronchial approach based on CT findings for the second specimen collection. For larger lesions, adjust the depth of specimen collection accordingly, and if adenocarcinoma is suspected, retain the same collection site.

The tumor cell counts collected by brushing increased with repeated brushings. This is evidenced by the reclassification of more than half of the patients from class 3 in the initial ROSE to class 4 in the final ROSE diagnosis. Additionally, the cumulative percentage of class 4 diagnoses increased with each subsequent brushing. The frequency of brushings should be determined based on ROSE results; however, brushing more than five times is not recommended because it only results in a slight increase in positivity. When performing NGS or PD-L1 testing on specimens collected during bronchoscopy, the higher the tumor cell count, the higher the success rate of the tests. Therefore, it is recommended to perform additional brushing to collect more cells, even if tumor cells are already present in the cytology specimen, to ensure the adequacy of the sample for NGS or PD-L1 testing. Further research is required to establish the precise tumor cell counts needed for successful NGS or PD-L1 testing of cytology specimens.

This study has some potential limitations. It was a single-center, retrospective study, which may have influenced the examiner's judgment regarding the presence or absence of ROSE, and selection bias may exist. However, the ROSE group did not have significantly more small lesions or pulmonary apex cases that were difficult to diagnose using bronchoscopy; hence, the possible effect was likely minimal. Prospective randomized multicenter trials are required to avoid potential bias.

In conclusion, the use of ROSE on brush cytology during bronchoscopy with EBUS-GS for PPL improved the diagnostic sensitivity of the procedure. In some cases, repeated brushings under ROSE converted the initial negative results to positive and increased the tumor cell counts in the brushing specimens.

## Data Availability

The datasets generated and analyzed during the current study are available from the corresponding author upon reasonable request.
